# Clinical outcomes of laminoplasty for patients with lysosomal storage disease including mucopolysaccharidosis and mucolipidoses: a retrospective cohort study

**DOI:** 10.1186/s13023-021-02031-9

**Published:** 2021-09-28

**Authors:** Hidetomi Terai, Koji Tamai, Masatoshi Hoshino, Hiromitsu Toyoda, Akinobu Suzuki, Shinji Takahashi, Yusuke Hori, Akito Yabu, Hiroaki Nakamura

**Affiliations:** Department of Orthopaedic Surgery, Osaka City University Graduate School of Medicine, 1-5-7, Asahimachi, Abenoku, Osaka, Osaka 545–8585 Japan

**Keywords:** Mucopolysaccharidoses, Mucolipidoses, Laminoplasty, Cervical, Myelopathy, Lysosome storage diseases

## Abstract

**Background:**

Although the clinical efficacy of laminoplasty in adult cervical spondylotic myelopathy or ossification of posterior longitudinal ligament has been frequently reported, there are only few reports of laminoplasty for patients with lysosome storage diseases (LSDs). Therefore, this study aimed to report the midterm clinical and radiological outcomes of patients with LSDs after cervical laminoplasty.

**Methods:**

Six patients with LSD who underwent laminoplasty with/without C1 laminectomy for cervical myelopathy were enrolled. Clinical evaluations, including the cervical Japanese Orthopedic Association (cJOA) score and visual analog scale (VAS) scores for upper extremity numbness, and radiographic parameters, including C2–C7 lordotic angle, atlanto-dens interval (ADI), and ⊿ADI, were evaluated preoperatively, at 2 years postoperatively, and at the final follow-up.

**Results:**

Five patients had mucopolysaccharidoses (type I: n = 1, II: n = 3, VII: n = 1) and one patient had mucolipidoses type III. The mean age of patients at surgery was 27.5 years, and the mean postoperative follow-up period was 61 months. All mucopolysaccharidoses cases required C1 posterior arch resection with C2–C7 laminoplasty. No critical complications were observed postoperatively. There were no significant differences in C2–C7 angle (p = 0.724) and ⊿ADI (p = 0.592) between the preoperative and final follow-ups. The cJOA score and VAS for numbness significantly improved at the final follow-up (p = 0.004 and p = 0.007, respectively).

**Conclusions:**

The cervical myelopathy in patients with LSD could be safely and effectively treated with laminoplasty with/without C1 posterior arch resection after excluding patients with atlantoaxial instability. Atlantoaxial stability and symptom improvement could be maintained at an average of 5 years postoperatively.

## Background

Mucopolysaccharidoses (MPS) and mucolipidoses (ML) comprise a group of rare inherited lysosomal storage diseases (LSDs). The incidence of MPS is 1.53 per 100,000 live births, although that of overall LSDs is one per 7000 individuals [1–3]. The accumulation of undegraded cholesterol, phospholipid, and glycosaminoglycan (GAG) substrates occurs in the cell lysosomes of various tissues due to insufficiency of the metabolic enzyme. Each type of MPS and ML displays a wide spectrum of clinical manifestations, including the onset of symptoms [4, 5]. The accumulation of storage materials in prenatal chondrocytes affects primary bone formation, which results in severe skeletal manifestations, secondary ossification centers, and the epiphyseal cartilage of growth plate. It disturbs the normal systemic endochondral and membranous bone growth after birth, which results in various skeletal impairments, known as multiplex dysostosis, including the spine [6–8].

For the past 20 years, two main medical treatments had been developed for patients with MPS and are currently available in clinical practice: hematopoietic stem cell transplantation (HSCT) and enzyme replacement therapy (ERT) [9–11]. In HSCT, healthy donor cells are transplanted, and the enzymes secreted by donor cells are then taken up by the recipient’s body through cross-correction. On the other hand, ERT delivers the specific recombinant enzyme that is deficient in the patient. As the prognosis of patients with LSDs is expected to prolong with these new therapies, improvement in the quality of life (QOL) of such patients could be the next challenge.

Spinal disorders and their severity vary depending on disease type and activity. Typical spinal manifestations among LSDs include atlantoaxial instability and cervical developmental spinal canal stenosis [12, 13]. Atlantoaxial instability is frequently observed in patients with MPS types I, IV, and VI. Cervical stenosis is widely recognized in most MPS types (including MPS types I, II, VI, and VII) and ML [14, 15]. Among patients with spinal disorders, the progression of cervical canal narrowing and its resultant cervical myelopathy are critical challenges in patients with LSDs because these directly lead to deterioration of the patients’ QOL. Therefore, patients who have cervical stenosis could require surgical intervention [13, 16, 17].

Laminoplasty is the established surgical technique to treat multilevel cervical stenosis, and it is widely performed for cervical spondylotic myelopathy (CSM) and ossification of posterior longitudinal ligament (OPLL) [18–20]. The advantages of cervical spine laminoplasty include preserving the cervical range of motion (ROM) and preventing postoperative kyphotic deformity by preserving posterior elements [21–23]. Although many studies have proven the clinical efficacy of laminoplasty in adult CSM or OPLL [19, 24, 25], only few reports of laminoplasty are available in the literature for patients with LSDs. Therefore, this study aimed to report the midterm clinical and radiological outcomes of patients with LSDs after cervical laminoplasty.

## Results

The demographic data of each patient, including disease type, age at surgery, presence of supportive therapies, clinical scores, and follow-up period after surgery, are summarized in Table 1. The mean age at surgery was 27.5 (13–38) years, and the mean follow-up period was 61 (30–108) months. Of the 6 patients, 4 were treated with ERT. The main stenotic level in 5 of the 6 patients was C1 (Table 2).

**Table 1:** Demographics of the six patients with LSDs

Patient no.	Disease type	Age at surgery (years)	Sex	Medical therapy	cJOA score (points)	VAS score (mm)	Follow-up (month)
#1	MLP type III	38	Female	–	12	20	72
#2	MPS type I	19	Male	ERT/ HSCT	14	70	48
#3	MPS type II	13	Male	ERT	16	60	108
#4	MPS type II	38	Male	ERT	11.5	60	48
#5	MPS type II	37	Male	ERT	12.5	10	30
#6	MPS type VII	20	Male	–	13	80	60

*LSDs* Lysosomal storage disease, *MLP* mucolipidosis, *MPS* mucopolysaccharidosis, *ERT* enzyme replacement therapy, *HSCT* hematopoietic stem cell transplantation, *cJOA* cervical Japanese Orthopedic Association

**Table 2:** Preoperative radiographic characteristic of the six patients with LSDs

Patient no.	C2/7 angle (degree)	ADI (mm)	⊿ADI (mm)	ADI of CT (mm)	ROM (degree)	Most stenotic level on MRI
#1	4	1	0.1	1.3	62	C3-4
#2	4	1	0.4	1.7	53	C1
#3	16	2.5	2	2.2	56	C1
#4	-1	1.6	1.4	1.6	54	C1
#5	2	1.3	0.2	1.4	56	C1
#6	39	1.9	1	2.4	69	C1

*ADI* Atlanto-dens interval, *ROM* range of motion

All patients with MPS required C1 posterior arch resection accompanied by C2-C7 laminoplasty, whereas the patient with ML was treated with C2-C7 laminoplasty without C1 posterior arch resection. No critical complication was observed after surgery in this cohort. Cervical ROM significantly decreased from 58.3°±13.7° preoperatively to 35.5° ± 10.7° at the final follow-up (p = 0.003). Meanwhile, the C2-C7 angle preoperatively and at the final follow-up (10.7°±13.7° vs. 14.0°±15.3°, p = 0.724, Table 3), atlanto-dens interval (ADI), and ⊿ADI (ADI: 1.6 ± 0.5 vs. 1.5 ± 0.4, p = 0.651, ⊿ADI: 0.9 ± 0.7 vs. 0.7 ± 0.4, p = 0.592) showed no significant differences. Computer tomography (CT) revealed bony fusion of the gutter at 12 months postoperatively in all patients. Magnetic resonance imaging (MRI) showed that the canal expansion remained after surgery. High-intensity changes on T2-weighted MRI scans were identified in 4 patients (cases 1, 4, 5, and 6) before surgery, which continued until the final follow-up. The progress of stenosis or instability caused by new accumulations or surgical traumas was not observed in any patient. Concerning the changes in clinical symptoms, the cervical Japanese Orthopedic Association (cJOA) score improved from 13.2 ± 1.5 preoperatively to 16.5 ± 0.8 at the final follow-up (p = 0.004, Table 4). The visual analog scale (VAS) scores of upper extremity numbness improved from 50.0 ± 25.8 preoperatively to 10.8 ± 10.2 at the final follow-up (p = 0.007).

**Table 3:** Postoperative radiographic change of the six patients with LSDs

Patient no.	ROM (degree)				C2-7 angle in neutral position			
	Preop	1 yr	2 yrs	FU	Preop	1 yr	2 yrs	FU
#1	62	16	26	23	4	15	11	11
#2	53	64	36	24	4	-5	-3	-3
#3	56	65	62	46	16	13	11	14
#4	54	39	34	29	-1	-12	-9	-2
#5	56	52	50	50	2	5	22	22
#6	69	46	48	41	39	23	21	42
Average	58.3 ± 13.7	47.0 ± 16.7	42.7 ± 11.9	35.5 ± 10.7	10.7 ± 13.7	6.5 ± 12.0	8.8 ± 11.5	14.0 ± 15.3
p-value				0.003				0.724

Patient no.	ADI (mm)				⊿ADI (mm)			
	Preop	1 yr	2 yrs	FU	Preop	1 yr	2 yrs	FU
#1	1	1	1	1	0.1	0	1	0.5
#2	1	1.5	1.5	1.5	0.4	0.5	0.5	0.5
#3	2.5	1.9	1.8	2	2	0.2	1.5	1.6
#4	1.6	1.2	1.5	1	1.4	0.8	1.1	0.4
#5	1.3	1.3	1.8	1.8	0.2	0.6	0.7	0.7
#6	1.9	2.4	1.9	1.4	1	0.9	1.5	0.6
Average	1.6 ± 0.5	1.6 ± 0.5	1.6 ± 0.3	1.5 ± 0.4	0.9 ± 0.7	0.5 ± 0.3	1.1 ± 0.4	0.7 ± 0.4
p-value				0.651				0.592

P-value was calculated as the comparison with preoperative values

*Preop* Preoperative, *yr* year, *yrs* years

**Table 4:** Postoperative clinical change of the six patients with LSDs

Patient no.	cJOA score				Numbness			
	Preop	1 yr	2 yrs	FU	Preop	1 yr	2 yrs	FU
#1	12	16	17	17	20	0	0	0
#2	14	17	17	17	70	30	25	30
#3	16	17	17	17	60	20	20	15
#4	11.5	16	16	16	60	20	10	10
#5	12.5	17	17	17	10	0	0	0
#6	13	14	15	15	80	10	10	10
Average	13.2 ± 1.5	16.2 ± 1.1	16.5 ± 0.8	16.5 ± 0.8	50.0 ± 25.8	13.3 ± 11.1	10.8 ± 9.3	10.8 ± 10.2
p-value				0.004				0.007

P-value was calculated as the comparison with preoperative values

*Preop* Preoperative, *yr* year, *yrs* years

### Representative cases

**Fig. 1:**
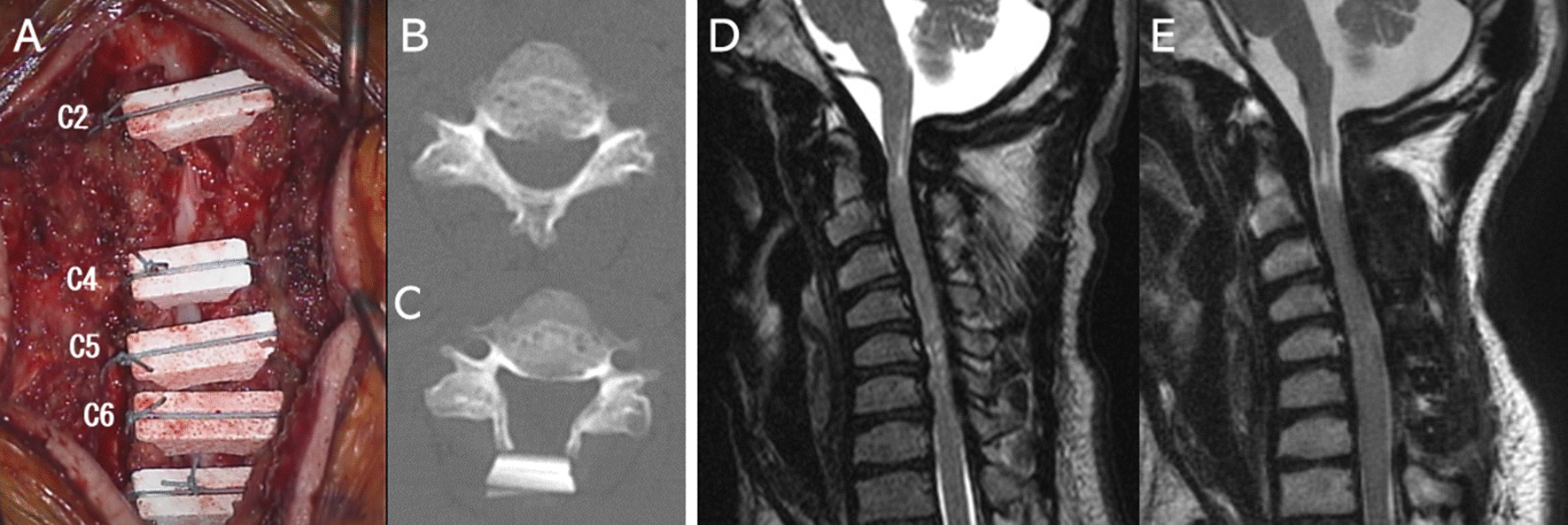
A 37-year-old male patient with MPS type II. Double door laminoplasty combined with C1 posterior arch resection and partial resection of the foramen magnum dorsal wall. **A** HA spacers were placed and ligated between the opened laminae. **B** Axial CT image of the lamina before operation. **C** Opened laminar and HA spacer (same level as **B**). **D** T2WI MR image of the cervical spine before operation. **E** T2WI MR image of the same patient at 6 months after surgery. Note that the cervical spinal canal was expanded. The high intensity area at the C1/2 level became more evident and that at the C5/6 level disappeared after surgery. * MPS* mucopolysaccharidoses, *HA* hydroxyapatite, *CT* computed tomography, *MR* magnetic resonance

A 37-year-old male patient with MPS type II (case #5) underwent double-door laminoplasty combined with C1 posterior arch resection and partial resection of the dorsal wall of the foramen magnum (Fig. 1). The cJOA score improved from 12.5 to 17 after surgery.

**Fig. 2:**
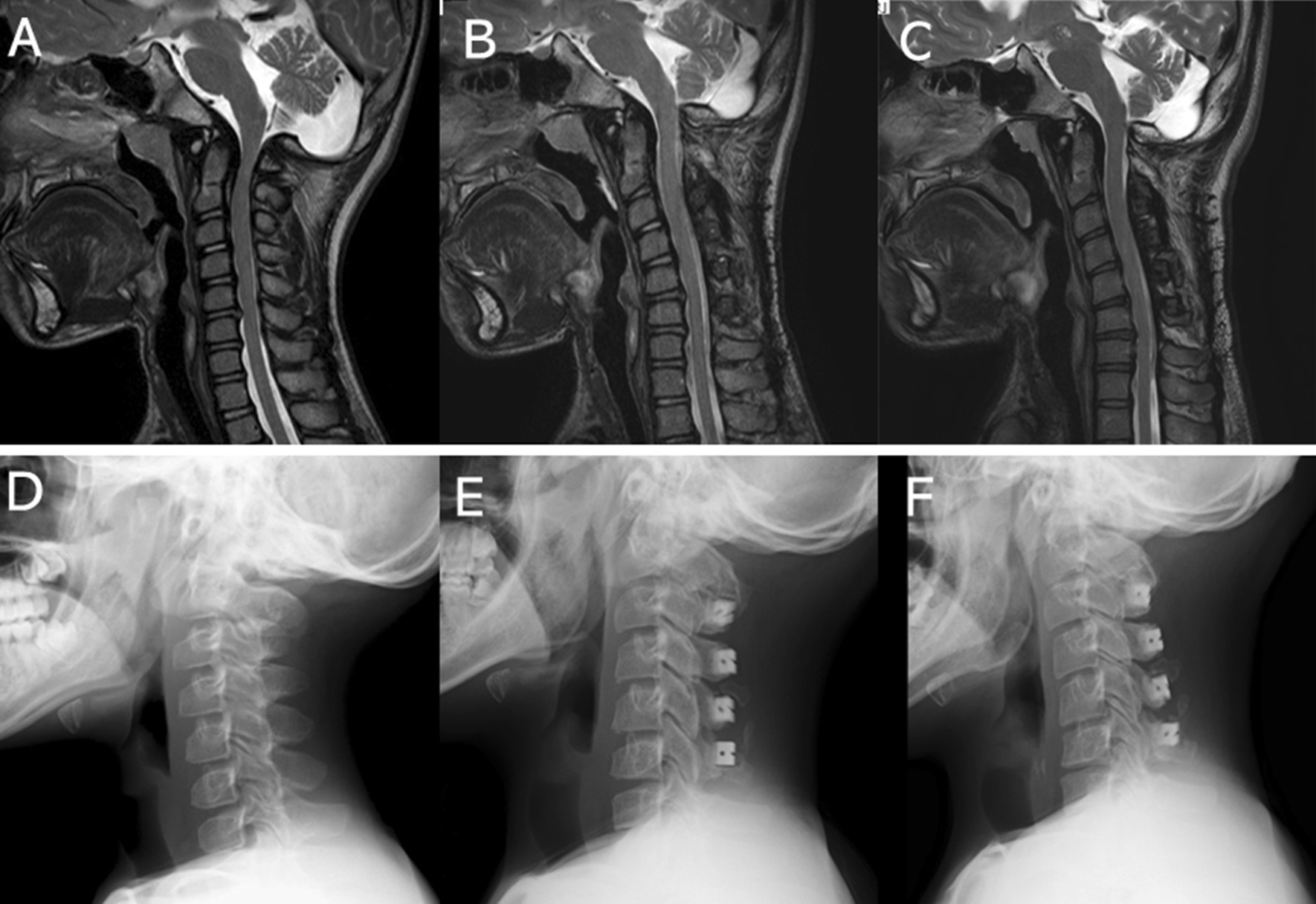
T2WI sagittal images and plain lateral radiographs of a 13-year-old male patient with MPS type II. The patient experienced bilateral hand numbness when his neck was extended. **A**, **D** Before operation. **B**, **E** Two years postoperatively. **C**, **F** Eight years postoperatively. Symptom disappeared postoperatively. Note that the cervical kyphosis gradually progresses in 8 years without clinical symptom exacerbation. *MPS* mucopolysaccharidoses

A 13-year-old male patient with MPS type II (case #3) underwent double-door laminoplasty combined with C1 posterior arch resection (Fig. 2).

**Fig. 3:**
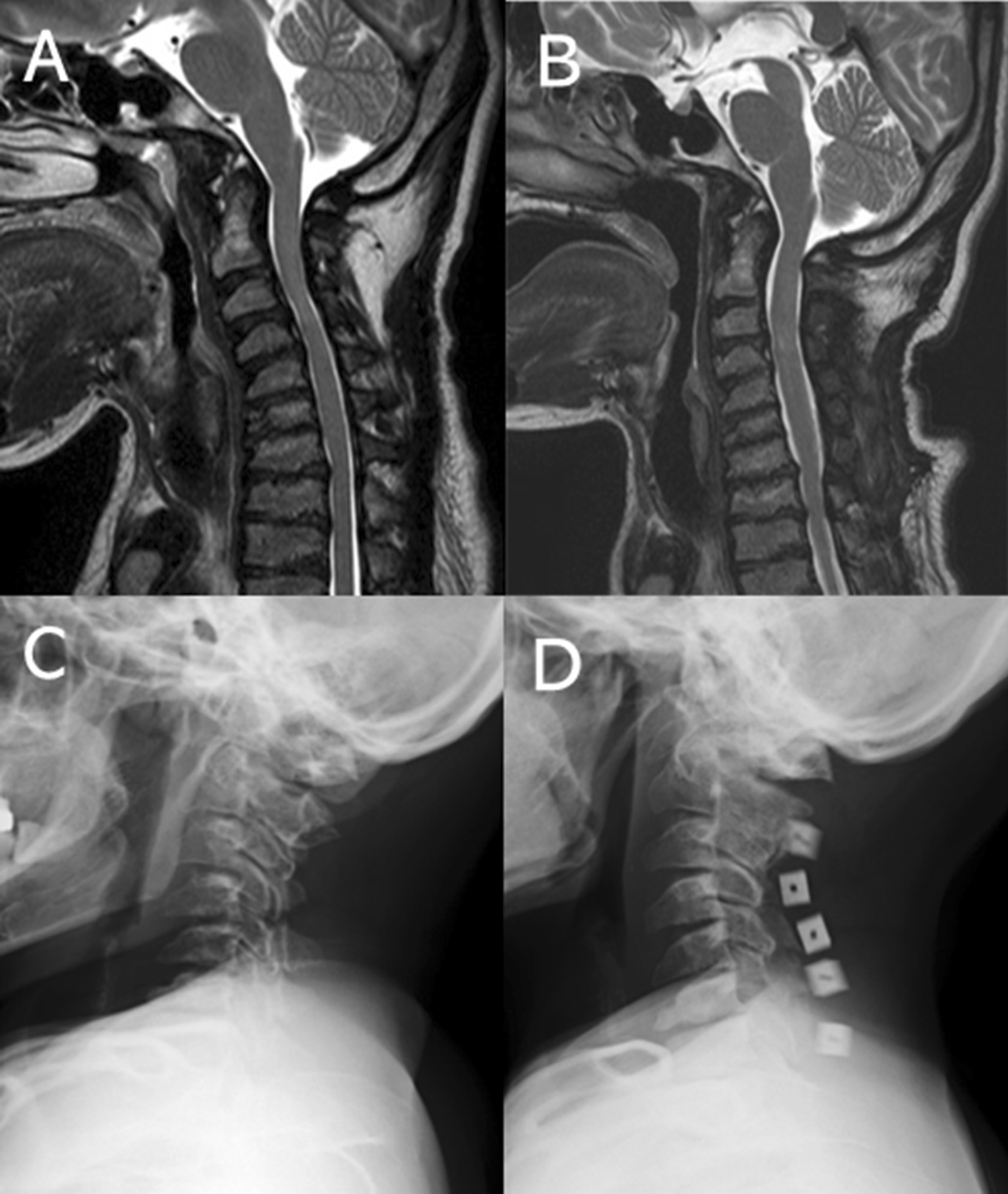
T2WI sagittal images and plain lateral radiographs of a 38-year-old female patient with ML type III. Her symptoms include clumsiness and gait disturbance. **A**, **C** Before operation. **B**, **D** Two years postoperatively. The sagittal cervical alignment became lordotic after surgery, which was maintained until the final follow-up (6 years postoperatively). Her myelopathic symptom disappeared immediately after surgery. *ML* mucolipidoses

A 38-year-old female patient with ML type III (case #1) underwent double-door laminoplasty from C2 to C7 (Fig. 3). Preoperative symptoms, including hand clumsiness and gait disturbance, disappeared immediately after surgery.

**Fig. 4:**
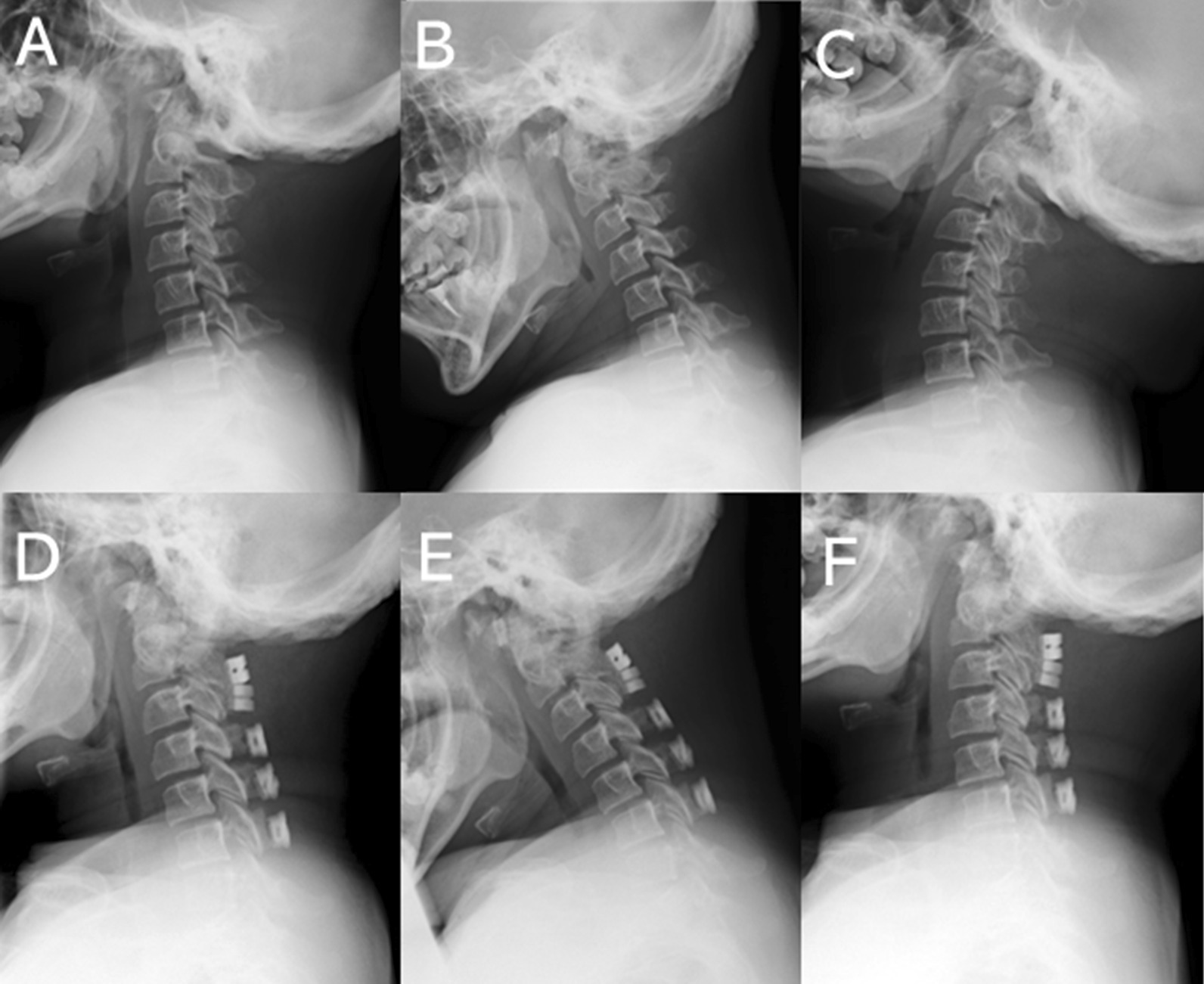
The cervical spine ROM after laminoplasty in a 19-year-old male patient with MPS type I. **A**–**C** Plain radiographs of the cervical spine before surgery at neutral, flexion (34˚), and extension (20˚) positions from the left. **D**–**F** Plain radiographs of the cervical spine at 4 years postoperatively at neutral, flexion (17˚), and extension (12˚) positions from the left. Approximately 50 % reduction of ROM was observed in this patient. * MPS* Mucopolysaccharidoses, *ROM* range of motion

A 19-year-old male patient with MPS type I (case #2) underwent double-door laminoplasty combined with C1 posterior arch resection (Fig. 4). Although the symptoms, mainly hand numbness, disappeared after surgery, the cervical ROM decreased significantly at the final follow-up.

## Discussion

Evaluation of the upper cervical level could be critical in establishing the surgical strategy for cervical myelopathy in patients with LSDs. The main stenotic cervical level of patients with MPS was C1 due to the hypoplasticity of the C1 lamina in our series; some types of LSDs are often accompanied by atlantoaxial instability [12, 13]. When atlantoaxial instability is evident, occipito-cervical fixation, rather than cervical laminoplasty, with C1 laminectomy should be indicated. Otherwise, the cervical myelopathy of patients with LSDs could be safely treated with laminoplasty with or without C1 posterior arch resection. In addition, the current study demonstrated that atlantoaxial stability and symptom improvement could be maintained at an average of 5 years postoperatively. The spinal cord compression at the upper cervical level could affect respiratory function and not only the movement of the whole extremity, but also the respiratory function. Because the main cause of death in patients with MPS is respiratory dysfunction, we strongly recommend surgically treating patients with LSDs at an earlier stage of myelopathy, such as stages when only numbness is a symptom of myelopathy.

Regarding the surgical method of laminoplasty, open-door and double-door laminoplasties are two major methods [26, 27]. The distinct features of LSDs from adult CSM/OPLL include the small characteristic shape of the spine, presence of accumulation in the extradural space, and non-requirement of extradural resection of the accumulation. Based on our institution’s surgical strategy, open-door laminoplasty is employed to treat adult patients with CSM/OPLL owing to the sufficient space available for spacers. However, for patients with LSDs, it is challenging to put spacers and treat extradural materials with a one-side opening of the laminae due to the hypertrophic deformity of the laminae (Fig. 1). In addition, age could be an important factor in selecting the surgical method. In our study, we excluded two patients, a 3-year-old boy with MPS II and a 1-year-old girl with ML type III, who underwent total laminectomy of the cervical spine from the current series, as their spines were very small to undergo laminoplasty.

One of the advantages of laminoplasty is ROM preservation and kyphotic change prevention. In this case series, the postoperative cervical ROM was significantly decreased compared with the preoperative ROM, according to previous reports analyzing patients with adult CSM/OPLL [21–23]. However, in our series, the average cervical ROM at an average of 5 years postoperatively was still 36°. As the cervical ROM is one important factor for achieving high QOL and preventing dysphagia [28], we recommend avoiding fusion surgery as much as possible. In addition, although patients with LSDs still have a much shorter life expectancy than the normal population, life expectancy could be prolonged owing to the development of patient care and interventions, such as ERT and HSCT [9–11]. A prolonged life induces degenerative changes in the joint and spine, which may require additional surgical interventions. Therefore, it is now significant to consider and introduce surgical methods to prevent degenerative changes, such as adjacent disc degeneration observed after fusion surgery.

Another advantage of cervical laminoplasty compared with cervical laminectomy is the low incidence of kyphotic change postoperatively. Machino et al. reported that nearly 8 % of patients are predisposed to cervical kyphosis [29]. Moreover, McGirt et al. reported that laminoplasty for the resection of intramedullary spinal cord tumor in children was associated with a decreased incidence of progressive spinal deformity requiring fusion compared with laminectomy [30]. In accordance with such studies, current results showed no significant change in the C2–C7 angle at an average of 5 years postoperatively.

The disadvantage of cervical laminoplasty is the limited indication for pediatric patients with LSDs, as their laminar size is too small to undergo laminoplasty and allow a spacer placement. In addition, although there are some types of spacers for cervical laminoplasty, all of them were designed for adult patients, and no spacers that could fit small pediatric patients were noted. Indeed, during the study period, we have two patients in whom laminectomy was performed, rather than laminoplasty, due to the small laminae. In addition, the surgical indication of cervical laminoplasty is limited for patients with cervical instability. For example, atlantoaxial instability due to odontoid hypoplasia in patients with MPS-IV has been well described in the literature [31, 32]. In this case series, we carefully evaluated the cervical instability using dynamic radiography or CT images and excluded the case with atlantoaxial instability.

Several limitations to the present study need to be addressed. Firstly, the retrospective nature of the study makes it difficult to exclude bias, especially regarding the referral for a certain postoperative rehabilitation program and the particular surgical techniques utilized. Secondly, the number of patients is relatively small and inconsistent disease type. In addition, this study is a case series, not a comparative study. All these limitations prevent us from making a definitive conclusion regarding the safety and clinical outcomes of surgical treatment for patients with LSDs. However, the possibility for spine physicians to treat such patients is significantly increasing owing to the current advancement in medical treatment. Therefore, we believe that our data could be useful for spine physicians when treating cervical myelopathy in patients with LSDs.

## Conclusions

In the current case series, we found that the cervical myelopathy of patients with LSDs could be safely treated with laminoplasty with or without C1 posterior arch resection after eliminating patients with atlantoaxial instability. Additionally, the current study confirmed that the atlantoaxial stability and improvement of symptoms could be maintained at an average of 5 years postoperatively.

## Methods

### Study design and ethics

We conducted a retrospective cohort study to report the midterm clinical and radiological outcomes of patients with LSDs after cervical laminoplasty. Written informed consent was obtained from all participants. The study protocol was approved by the Institutional Review Board of our institution (No. 3170). No funds were received in support of this work. No benefits in any form have been or will be received from a commercial party directly or indirectly related to the subject of this manuscript. All procedures were performed in accordance with the Declaration of Helsinki and the Ethical Guidelines for Medical and Health Research Involving Human Subjects in Japan.

### Patient population

From December 2011 to March 2018, six patients with MPS (type I: 1, II: 3, VII: 1) or MLS type III underwent laminoplasty with or without C1 laminectomy for cervical myelopathy by a single surgeon in our orthopedic surgery department. All patients underwent MRI, CT, and dynamic cervical radiography preoperatively to evaluate the level of stenosis and segmental instability. One patient with atlantoaxial instability, defined as an ADI of > 3 mm measured on preoperative neutral radiography or CT images, was treated with fusion surgery and excluded from the current case series. Moreover, two patients aged ˂ 3 years were excluded as their laminae were too small to undergo laminoplasty; thus, they were treated with laminectomy instead. General conditions and airway status were carefully evaluated by a pediatrician and an anesthesiologist before the induction of general anesthesia. All patients required fiberoptic intubation due to airway management difficulty related to the narrowing of the oropharynx and airway or trismus.

### Surgical technique

Surgical methods of cervical laminoplasty are broadly divided into two types of osteotomy method to open the laminar: open-door type and double-door type [26, 27]. In the open-door type, an osteotomy is performed at one side of the lamina-facet junction. Meanwhile, in the double-door type, an osteotomy is performed at the central spinous process and lamina. Although each method has its advantages and disadvantages, its common purpose is to expand the narrowed spinal canal and afford the opportunity of reactivating the spinal cord [33]. In this series, we adopted the double-door laminoplasty due to the spacer size (see Discussion section).

Patients were carefully positioned prone while keeping the neck in a neutral position after fixing the head with a MAYFIELD fixator. Skull bone thickness was checked by CT scan before the MAYFIELD fixator was applied. For patients aged < 10 years or those with a thin skull, we used a halo vest instead of using the MAYFIELD fixator [34]. Intraoperative neurophysiological monitoring is recommended for the spinal surgery of LSDs [35]. A midline incision from the occipital to the T1 lamina process is created. Paraspinal muscles, including the semispinalis, are detached with a tiny bone chip and exposed laterally from the laminae. A midline cut is made; then, gutters are made on both sides inside the facet joint to leave a thin inner cortex to make hinges using a high-speed drill. This process is completed from C2 to C7. The dorsal wall of the foramen magnum is partially resected as necessary. As for C1, the posterior arch is resected until the lateral edge of the dura mater is visualized, followed by the subtotal resection of the atlanto-occipital membrane. The LSD-related accumulated materials are located in the extra-dural space between the dura mater and yellow ligaments. Hard and elastic accumulated materials on the dura mater should be carefully resected and removed to achieve adequate decompression. Durotomy or duroplasty is not required because the accumulation was outside the dura mater, although they are recommended in previous reports [34, 36]. After confirming dural pulsation, hydroxyapatite spacers are set to bridge the opened laminae on both sides. A drainage tube is placed after repeated saline irrigation. Postoperative care includes obligatory intensive care monitoring because of the high risk of respiratory/cardiac insufficiency in LSDs. Patients are mobilized freely without a brace because their neck lengths are too short to make adequate cervical collars. Patients are followed up clinically with MRI and functional cervical radiography.

### Clinical evaluation

Clinical evaluations were performed preoperatively, at 3 months, 1 year, and 2 years postoperatively, and at the final follow-up. The cJOA score, a physician-assessed score used to evaluate the severity of cervical myelopathy, was determined [37]. The VAS for upper extremity numbness was recorded as the component of the patient-oriented score [38]. The VAS scores were evaluated using a 100-mm long horizontal line with extremes indicated as “no symptoms: 0” and “worst symptoms imaginable: 100”. The patients marked the point on the line representing their perception of their current state.

### Radiographic evaluation

Radiographic parameters were evaluated using preoperative and postoperative cervical plain radiographs. Each parameter was defined as follows: C2-C7 lordotic angle, the lordotic angle between the tangent lines of the lower endplates of C2 and lower endplates of the C7 vertebral body; ROM, defined as the difference in C2-C7 lordotic angles between the maximum flexion and extension positions [39]; ADI, the horizontal distance between the anterior arch of the atlas and the dens of the axis, and ADI was evaluated using weight-bearing radiographs at neutral, flexion, and extension positions and preoperative CT image at neutral position; and ⊿ADI, the differences in ADI on weight-bearing radiographs between flexion and extension positions. Observers reviewed the images and measured the parameters using computer software (Synapse; Fujifilm, Tokyo, Japan). The bone union of the gutter was evaluated using the CT scan taken within 12 months postoperatively, and the signal change of the spinal cord was evaluated using T2-weighted images of preoperative and postoperative MRI [40].

### Statistical analysis

Average values were presented as average ± 1.0 standard deviation or range from minimum to maximum values. The values of the continuous variables, such as ROM, C2–C7 angle, ADI, and ⊿ADI, obtained preoperatively and at the final follow-up were compared using a paired t-test. In addition, the cJOA and VAS scores of upper extremity numbness obtained preoperatively and at the final follow-up were compared using a paired t-test. All analyses were performed using SPSS software (version 23; SPSS, Chicago, IL). A p-value < 0.05 was considered statistically significant.

## Data Availability

The datasets generated and/or analyzed in the current study are available from the corresponding author on reasonable request.

## Abbreviations

ADI: Atlanto-dens interval
cJOA: Cervical Japanese Orthopedic Association
CSM: Cervical spondylotic myelopathy
CT: Computer tomography
ERT: Enzyme replacement therapy
GAG: Glycosaminoglycan
HSCT: Hematopoietic stem cell transplantation
LSDs: Lysosome storage diseases
ML: Mucolipidoses
MPS: Mucopolysaccharidoses
MRI: Magnetic Resonance Imaging
OPLL: Ossification of posterior longitudinal ligament
QOL: Quality of life
ROM: Range of motion
VAS: Visual analog scale
